# Autoantibodies against P29ING4 are associated with complex regional pain syndrome

**DOI:** 10.1007/s12026-020-09114-y

**Published:** 2020-02-01

**Authors:** N. T. Baerlecken, R. Gaulke, N. Pursche, T. Witte, M Karst, M. Bernateck

**Affiliations:** 1 Cologne, Germany; 2 Trauma Department, Medical University Hannover, Hannover, Germany; 3 Department of Clinical Immunology and Rheumatology, Medical University Hannover, Hannover, Germany; 4 Department of Anesthesiology, Pain Clinic, Medical University Hannover, Hannover, Germany

**Keywords:** CRPS, Autoantibodies, p29ING4, p29ING4 IC, Morbus Sudeck

## Abstract

**Introduction:**

Complex regional pain syndrome (CRPS) is a complication following trauma or surgery and may be difficult to diagnose since biomarkers are lacking. Using protein array technology, we found antibodies binding to p29ING4, which we further characterized using ELISA.

**Methods:**

Thirty-six sera of early-stage type 1 CRPS, 66 sera of rheumatoid arthritis (RA), 53 sera of axial spondyloarthritis (axSpA), 29 sera of psoriatic arthritis (PsA), 22 sera of patients after radial fractures (trauma control), and 100 sera of blood donors (BD) were analyzed for anti-p29ING4. We established ELISAs with 7 different antigens and using different secondary antibodies binding to IgG, IgG1, IgG2, IgG3, IgG4, IgA, and IgM, and 2 different tests to detect immune complexes (IC) of p29ING4 and IgG or IgG1.

**Results:**

The highest likelihood ratios versus CRPS and trauma control were observed considering the A1-23 (sensitivity 19%, specificity 100%, LR > 19) using IgG as a secondary antibody, the A120-165 (sensitivity 17%, specificity 100%, LR = 17) using IgG as a secondary antibody and the A120-165 (sensitivity 31%, specificity 95%, LR = 6.2) using IgA as a secondary antibody. IC of p29ING4 and IgG were present in 11/36 (31%) CRPS sera, 17/64 (27%) RA sera, 13/53 (25%) SpA sera, 5/29 (17%) PsA sera, 1/22 (5%) trauma control sera, and 4/100 (4%) sera of BD. IC of p29ING4 and IgG1 were present in 14/36 (39%) CRPS sera, 19/64 (30%) RA sera, 13/53 (25%) SpA, 1/29 (3%) PsA, 2/22 (9%) trauma control, and 4/100 (4%) of the BD sera.

**Conclusion:**

Due to the lack of other biomarkers of type 1 CRPS, P29ING4 autoantibodies could be helpful in its diagnostic work-up.

## Introduction

Complex regional pain syndrome (a.k.a. CRPS, Sudeck disease, sympathetic reflex dystrophy) is a frequent complication following trauma or operation with a prevalence of about 30/100.000 [1]. Prospective studies in patients with distal radial fractures revealed an incidence of this complication of up to 20% [1–4]. CRPS has a relevant socioeconomic impact, because it frequently causes severe longstanding disability in afflicted patients.

The current diagnostic guidelines distinguish between CRPS without (CRPS type 1) or with obvious nerve lesion (CRPS type 2) [5].

As CRPS is a clinical diagnosis, the characteristic clinical symptoms have to be recognized and differentiated from ordinary posttraumatic or postoperative findings and by the existence of other conditions or diseases. During the early phase following trauma, this discrimination is difficult because of similarities between CRPS and injury-related symptoms [2]. Thus, the first diagnosis of CRPS is made in many cases with a delay of months or even years after the onset of symptoms in some patients. Evidence suggests however that the benefit of treatment is best, when it is started early [5, 6].

In addition to sympathetic dysfunction, a neurogenic inflammation at the site of the affected limb may be one of the main underlying pathogenic mechanisms of type 1 CRPS [7, 8]. Cytokines, particularly tumor necrosis factor (TNF)-alpha, seem to play an important role in the mediation of mechanical hyperalgesia in type 1 CRPS and other neuropathic pain syndromes [9–16].

Autoimmune etiology of CRPS is a new pathophysiological concept and may have a severe impact on diagnostics and treatment of this chronic disease. Interestingly, Blaes and colleagues detected surface-binding autoantibodies in a subgroup of type 1 and type 2 CRPS patients [17, 18]. These antibodies were directed against antigens of the autonomic nervous system, as shown by the reactivity of patient sera with sympathetic and myenteric plexus neurons as well as cholinergic neuroblastoma cells. The origin of the antibodies was unknown, and their specificity was not established. It was also not known whether the antibodies were already present before the trauma. The presence of autoantibodies could be an additional diagnostic tool or a target for immunosuppressive treatment, e.g., TNF-alpha inhibition or rituximab.

In summary, there is a need for CRPS specific antibody-test procedures to support the differential diagnostics of CRPS and other posttraumatic complications. Therefore, we used protein array technology for screening of autoantibodies in CRPS type 1 and identified p29ING4 as an antigen. The p29ING4 belongs to a family of tumor suppressor proteins that can influence tumor protein 53 (TP53) and therefore inactivate cell growth and activate apoptosis. It is present in all human cells. The p29ING4 interacts with TP53 and EP300/p300 via its PHD-finger domain. This leads to the upregulation of p65 and NF-kappa alpha, which might link to its possible interaction to inflammation [19].

For larger evaluation of controls, we established different ELISAs. We investigated their diagnostic potential as early markers at disease onset.

## Methods

### Sample collection

We collected sera of patients with early-stage type 1 CRPS (*n* = 36) and patients after trauma of the distal forearm and the hand without any clinical sign for CRPS (*n* = 22), patients with rheumatoid arthritis (RA, *n* = 64), patients with axial spondyloarthritis (axSpA, *n* = 53), patients with psoriatic arthritis (PsA, *n* = 29), patients with febrile infectious diseases without underlying chronic infection (FID CI−, *n* = 68), patients with infectious diseases with underlying chronic infection (FID CI+, *n* = 44), and blood donors (BD, *n* = 100). The study was approved by our local ethical committee (project number 4928), and the patients provided informed consent.

CRPS was defined according to the Budapest Criteria 2007, RA according to the ACR/EULAR criteria 2010, axSpA according to the ASAS criteria, and PsA according to the CASPAR criteria [5, 20–22].

Demographic, laboratory, and disease activity data were obtained in the Department of Immunology and Rheumatology and the Department of Pain Medicine and the Trauma Department of the Medical University Hannover.

Clinical data included age, gender, acute phase reactants, serology, IASP-criteria characteristics, DAS28, BASDAI, physical examination, imaging, and treatment.

All samples were stored at − 20 °C for up to 2 years. In the case of patients with CRPS and patients with radial fracture, the unaffected arm was used for taking the blood samples.

### Laboratory

#### Protein array

Six different sera of patients with early-stage type 1 CRPS were screened for the presence of autoantibodies by high-density protein array (hEx1, Source BioScience LifeSciences, Germany). The method has been described in our former publication [23].

#### ELISA

##### Detection of autoantibodies against inhibitor of growth protein 4 (p29ING4)

We used the following peptides of the p29ING4 sequence as antigens:

A1-23 MAAGMYLEHYLDSIENLPFELQR

A24-44 NFQLMRDLDQRTEDLKA EID

A45-70 KLATEYMSSARSLSSEEKLALLKQIQE

A71-93 AYGKCKEFGD DKVQLAMQTY EMV

A94-114 DKHIRRLDTDLARFEADLKEK

A120-165 YDSSSSKGKKKGRTQKEKKAARARSKGKNS DEEAPKTAQKKLKLV recombinant partial length protein of p29ING4

The peptides were chemically synthesized (Biomatik, Wilmington, DE, USA) and their purity ranged between 97 and 99%.

The recombinant partial protein of p29ING4 contained the amino acid sequence (Aa) 109-225 of p29ING4. Its purity was > 90% by SDS-PAGE and it was derived from an *Escherichia coli* host expression system (Source BioScience LifeSciences, Germany).

For performing the ELISA tests, 96 well plates (Maxisorb, Nunc, Denmark) were coated with 1 μg human synthetic peptide of p29ING4 per well or with 0.2 μg p29ING4 protein diluted phosphate saline buffer (PBS) overnight. Then the plates were blocked with 300 μl/well Aesku sample buffer (Aesku.Diagnostics, Wendelsheim, Germany) per well for 30 min at room temperature (RT). The plates were incubated with 100 μl diluted sera (1:100) in the Aesku sample buffer for 30 min at RT. After 30 min of incubation, the plates were washed 3 times with 300 μl 0.05% Tween-20 PBS (PBS-T) (Thermo Fisher Scientific, DK).

Next, 100 μl/well of a secondary peroxidase (HRP)-goat anti-human antibody was added, which was diluted in the Aesku sample buffer.

The following antibodies were used:

IgG Fc HRP (Jackson ImmunoResearch Europe Ltd., Suffolk, UK), dilution of 2:10000

IgA HRP (Jackson ImmunoResearch Europe Ltd., Suffolk, UK) dilution of 5:10000

IgM HRP (Jackson ImmunoResearch Europe Ltd., Suffolk, UK) dilution of 5:10000

IgG1 (Abcam, Cambridge, UK) dilution of 1:2000

IgG2 (Abcam, Cambridge, UK) dilution of 1:2000

IgG3 (Abcam, Cambridge, UK) dilution of 1:2000

IgG4 (Abcam, Cambridge, UK) dilution of 1:1000

All antibodies were diluted 30 min before adding. The plates were incubated for 30 min at room temperature and washed 3 times with PBS-T. The color reaction was performed with TMB (Thermo Fisher Scientific, DK) for up to 30 min according to the manufacturer’s instructions and the ODs were read at 450 nm in an ELISA reader.

Detection of the inhibitor of growth protein 4 (p29ING4) immune complexes with IgG or IgG1

For performing the ELISA tests, maxisorb 96 well plates (Nunc, Denmark) were coated with 10 μl mouse monoclonal antibody against human p29ING4 (Santa-Cruz Biotechnology, Dallas, TX, USA) diluted in 10.5 ml PBS (100 μl/well) overnight. Then the plates were blocked with 300 μl/well PBS and 1% rabbit serum (Gibco, Life Technologies, Rockville, MD, USA) PBS per well for 30 min at room temperature (RT). After 30 min of incubation, the plates were washed 3 times with 300 μl 0.05% Tween-20 PBS (PBS-T) (Thermo Fisher Scientific, DK).

Next, 100 μl/well of a secondary HRP-goat anti-human antibody was added, which diluted in 1% rabbit serum PBS.

The following antibodies were used:

IgG H+L HRP (Jackson ImmunoResearch Europe Ltd., Suffolk, UK), dilution of 1:25000

IgG1 (Abcam, Cambridge, UK) dilution of 1:5000

The plates were incubated for 30 min at room temperature and washed 3 times with PBS-T. The color reaction was performed with TMB (Thermo Fisher Scientific, DK) for up to 30 min according to the manufacturer’s instructions and the optical density (OD) was read at 450 nm in an ELISA reader.

The cut-offs (CO) of the ELISAs were chosen by receiver operating characteristic (ROC) curve analysis. We defined patients with CRPS as the fraction of true positives out of the positives (true positive rate, TPR) and patients after trauma as the fraction of the false positives out of the negatives (false positive rate, FPR). We used 8 samples with different levels as standard and 8 blood donors as a negative control for all ELISAs.

### Statistic evaluation

Fisher’s exact test was used to evaluate differences in considered items within a group. Correlations were analyzed in terms of Spearman’s correlation. All *p* values refer to two-tailed tests. Two-tailed *p* < 0.05 was regarded as statistically significant.

The diagnostic significance of the tests was assessed by ROC curve analysis, and the areas under the curve (AUC) were evaluated using GraphPad Prism 5 software (GraphPad, La Jolla, CA, USA).

Using SPSS (IBM Corp. Released 2013. IBM SPSS Statistics for Windows, Version 22.0. Armonk, NY: IBM Corp.), we performed multivariate analysis comparing the positive samples of RA, SpA, and CRPS, and considering positive and negative patients with RA.

## Results

### Patients

#### CRPS

The median age was 55 (range 22–78) years (yrs). Seventeen patients were males and 19 were females. The mean of C-reactive protein (CRP) was 3 mg/l with a standard deviation (SD) of 3.2 mg/l. CRP was not elevated in these patients. The mean disease duration (DD) was 2.5 months (ms) with a SD of 1.9 ms. None of these patients received disease-modifying anti-rheumatic drugs (DMARDs) or glucocorticoid (GC) treatment.

#### Trauma control

Patients with a previous trauma had a median age of 39 (range 16–80) yrs and included 12 males and 10 females. The blood was taken within the first 3 months after the trauma.

#### RA

A total of 50/64 patients were females. The median age was 60 (range 22–80) yrs. The median DD was 13 yrs (range 1–32 yrs). A total of 7/64 patients had neither RF nor ACPA. The mean of the activity score DAS28 was 3.2 ± 1,8, of ESR 17 mm ± 29 mm and of CRP 4 mg/l ± 44 mg/l. A total of 39/64 patients received GC treatment, 46/64 conventional DMARDs, and 30/64 biological DMARDs.

#### AxSpA

The median age of the patients with axSpA was 48 yrs (range 23–65 yrs). A total of 32/53 were females. A total of 26/53 were HLA-B27 positive. A total of 32/53 could be classified as ankylosing spondylitis. The median DD was 16 (range 1–38) years. The mean BASDAI was 5.2 ± 2.3 and mean CRP level was 3.2 mg/l ± 12.2 mg/l.

#### Psoriatic arthritis

The median age of patients with PsA was 50 yrs (range 34–68 yrs). A total of 19/29 were females. The median DD was 12.5 (range 2–29) yrs. The mean CRP level was 3.5 mg/l ± 4,9 mg/l.

#### FID CI−

The median age of these patients was 54 yrs (range 18–84 yrs). A total of 38/68 were females. The mean CRP level was 96.8 mg/l ± 105.1 mg/l.

#### FID CI+

The median age was 44 yrs (range 29–85 yrs). A total of 6/44 were females. A total of 37/44 were infected with HIV, 8/44 with hepatitis B, and 6/44 with hepatitis C. A total of 10/44 of these patients were infected with both HIV and hepatitis B or hepatitis C. The mean CRP level was 92.1 mg/l ± 101.9 mg/l.

#### Blood donors

The blood donors had a median age of 42 (range 18–60) yrs. Fifty-three were males and 47 were females.

## Protein array results

The sera of each patient with CRPS bound between 5 and 20 different autoantigens. Less than five autoantigens were shared by more than 2 patients. However, we could detect p29ING4 antibodies in 4/6 patients with CRPS. Taking arrays of our previous tests of different inflammatory diseases and healthy donors as controls [23, 24], p29ING4 antibodies have a frequency of 3/12 patients with rheumatoid arthritis, 2/8 patients with axial spondyloarthritis, and 0/4 blood donors.

## Evaluation of different ELISA detection methods

Using the partial length recombinant protein of p29ING4 and the synthetic peptides A1-23, A24-44, A45-70, A71-93, A94-114, and A120-165, and p29ING4 IC, we tested 26 early-onsets CRPS and 22 patients after trauma without any clinical sign of CRPS by using different secondary antibodies against human IgG, IgA, IgM, IgG1, IgG2, IgG3, and IgG4 (see Table 1).

**Table 1:** The frequency of the partial length recombinant protein of p29ING4 and the synthetic peptides A1-23, A24-44, A45-70, A71-93, A94-114, and A120-165, and p29ING4 immune complexes in 26 patients with complex regional pain syndrome type I (CRPS) listed at the top of the table using different secondary antibodies against human IgG, IgA, IgM, IgG1, IgG2, IgG3, and IgG4. On the left side, four different cut-offs are listed 0/22, 1/22, 2/22, and 3/22 patients with distal radial fracture without CRPS/ trauma controls (T)

	Frequency in CRPS							
	A1-23	A24-44	A44-70	A71r3	A94-114	A120-165	p291 NG4 protein	IC
Cut-offs								
IgG								
0/22 T	5/26 (19%)	0/26 (0%)	0/26 (0%)	0/26 (0%)	0/26 (0%)	5/26 (19%)	1/26 (4%)	5/26 (19%)
1/22 T	5/26 (19%)	0/26 (0%)	0/26 (0%)	0/26 (0%)	2/26 (8%)	5/26 (19%)	3/26 (12%)	7/26 (27%)
2/22 T	6/26 (23%)	1/26 (4%)	2/26 (8%)	4/26 (15%)	4/26 (15%)	6/26 (23%)	6/26 (23%)	8/26 (31%)
3/22 T	7/26 (27%)	2/26 (8%)	2/26 (8%)	8/26 (31%)	5/26 (19%)	7/26 (27%)	8/26 (31%)	11/26 (42%)
IgG1								
0/22 T	1/26 (4%)	0/26 (0%)	0/26 (0%)	0/26 (0%)	0/26 (0%)	0/26 (0%)	0/26 (0%)	2/26 (8%)
1/22 T	2/26 (8%)	0/26 (0%)	0/26 (0%)	1/26 (4%)	1/26 (4%)	0/26 (0%)	1/26 (4%)	**5/26 (19%)**
2/22 T	5/26 (19%)	2/26 (8%)	2/26 (8%)	2/26 (8%)	5/26 (19%)	8/26 (31%)	3/26 (12%)	8/26 (31%)
3/22 T	5/26 (19%)	2/26 (8%)	4/26 (15%)	3/26 (12%)	6/26 (23%)	11/26 (42%)	5/26 (19%)	11/26 (42%)
IgG2								
0/22 T	1/26 (4%)	0/26 (0%)	0/26 (0%)	0/26 (0%)	1/26 (4%)	0/26 (0%)	1/26 (4%)	0/26 (0%)
1/22 T	4/26 (15%)	1/26 (4%)	2/26 (8%)	1/26 (4%)	2/26 (8%)	3/26 (12%)	2/26 (8%)	0/26 (0%)
2/22 T	5/26 (19%)	2/26 (8%)	4/26 (15%)	3/26 (12%)	6/26 (23%)	7/26 (27%)	2/26 (8%)	2/26 (8%)
3/22 T	7/26 (27%)	2/26 (8%)	4/26 (15%)	3/26 (12%)	6/26 (23%)	8/26 (31%)	5/26 (19%)	2/26 (8%)
IgG3								
0/22 T	0/26 (0%)	0/26 (0%)	0/26 (0%)	0/26 (0%)	0/26 (0%)	0/26 (0%)	0/26 (0%)	4/26 (15%)
1/22 T	2/26 (8%)	1/26 (4%)	1/26 (4%)	2/26 (8%)	2/26 (8%)	2/26 (8%)	2/26 (8%)	4/26 (15%)
2/22 T	4/26 (15%)	2/26 (8%)	2/26 (8%)	2/26 (8%)	2/26 (8%)	4/26 (15%)	2/26 (8%)	4/26 (15%)
3/22 T	4/26 (15%)	4/26 (15%)	2/26 (8%)	4/26 (15%)	4/26 (15%)	4/26 (15%)	5/26 (19%)	5/26 (19%)
IgG4								
0/22 T	4/26 (15%)	0/26 (0%)	0/26 (0%)	0/26 (0%)	0/26 (0%)	0/26 (0%)	3/26 (12%)	0/26 (0%)
1/22 T	4/26 (15%)	2/26 (8%)	0/26 (0%)	0/26 (0%)	2/26 (8%)	1/26 (4%)	3/26 (12%)	0/26 (0%)
2/22 T	4/26 (15%)	2/26 (8%)	2/26 (8%)	2/26 (8%)	5/26 (19%)	2/26 (8%)	3/26 (12%)	4/26 (15%)
3/22 T	4/26 (15%)	2/26 (8%)	2/26 (8%)	2/26 (8%)	6/26 (23%)	3/26 (12%)	4/26 (15%)	4/26 (15%)
IgA								
0/22 T	1/26 (4%)	0/26 (0%)	0/26 (0%)	0/26 (0%)	1/26 (4%)	0/26 (0%)	1/26 (4%)	0/26 (0%)
1/22 T	5/26 (19%)	1/26 (4%)	2/26 (4%)	1/26 (4%)	2/26 (8%)	3/26 (12%)	2/26 (8%)	0/26 (0%)
2/22 T	5/26 (19%)	2/26 (8%)	2/26 (4%)	2/26 (8%)	5/26 (19%)	**11/26 (42%)**	7/26 (27%)	2/26 (8%)
3/22 T	7/26 (27%)	2/26 (8%)	4/26 (15%)	3/26 (12%)	6/26 (23%)	12/26 (50%)	8/26 (31%)	2/26 (8%)
IgM								
0/22 T	0/26 (0%)	0/26 (0%)	0/26 (0%)	0/26 (0%)	0/26 (0%)	0/26 (0%)	0/26 (0%)	0/26 (0%)
1/22 T	2/26 (8%)	0/26 (0%)	0/26 (0%)	0/26 (0%)	0/26 (0%)	0/26 (0%)	1/26 (4%)	0/26 (0%)
2/22 T	2/26 (8%)	1/26 (4%)	1/26 (4%)	2/26 (8%)	2/26 (8%)	2/26 (8%)	2/26 (8%)	2/26 (8%)

Due to the results, we evaluated additional patients with CRPS, SpA, PsA, RA, FID CI−, FID CI+, and BD for IgG anti-A1-23, IgG anti-A120-165, IgA anti-A120-165, P29ING4 IgG IC, and p29ING4 IgG1 IC (shown in Tables 1 and 2).

**Table 2:** The frequency of IgG anti-A1-23, IgG anti-A120-165, IgA anti-A120-165, p29ING4 IgG IC, p29ING4 IgG1 IC, and their combined frequency in patients with complex regional pain syndrome type I (CRPS), trauma controls, patients with rheumatoid arthritis (RA), patients with axial spondyloarthritis (axSpA), psoriatic arthritis (PsA), patients with febrile infectious diseases and underlying chronic infection (FID CI+), patients with febrile infectious diseases and without underlying chronic infection (FID CI−), and blood donors (BD)

	CRPS	RA	SpA	PsA	FID CI+	FID CI−	Trauma control	BD
Anti-A1-23 IgG	7/36 19%	9/64 14%	11/53 21%	4/29 14%	28/44 64%	34/68 50%	0/20 0%	2/100 2%
Anti-A120-165 IgG	6/36 17%	12/64 19%	9/53 17%	3/29 10%	23/44 53%	26/68 38%	0/20 0%	2/100 2%
Anti-A10-165 IgA	15/36 42%	8/64 13%	4/53 8%	3/29 10%	22/44 50%	22/68 32%	2/22 9%	4/100 4%
P29ING4 IgG IC	11/36 31%	17/64 27%	13/53 25%	5/29 17%	5/44 11%	3/68 4%	1/22 5%	4/100 4%
P29ING4 IgG1 IC	14/36 39%	19/64 30%	13/53 25%	1/29 3%	11/44 25%	6/68 9%	2/22 9%	4/100 4%
Combination of both IC tests	16/36 44%	28/64 44%	16/53 30%	5/29 17%	13/44 30%	9/68 13%	3/22 14%	8/100 8%
Combination of IgG IC, anti-A120-165 IgA and IgG	25/36 69%	28/64 44%	16/53 30%	6/29 21%	29/44 66%	31/68 46%	2/22 9%	8/100 8%

The highest likelihood ratios versus CRPS and trauma control could be observed considering A1-23 (sensitivity 19%, specificity 100%, LR > 19), A120-165 (sensitivity 17%, specificity 100%, LR = 17) using IgG as secondary antibody, A120-165 (sensitivity 42%, specificity 91%, LR = 4.7) using IgA as secondary antibody, IgG p29ING4 IC (sensitivity 31%, specificity 95%, LR = 6.2), and IgG1 p29ING4 IC (sensitivity 39%, specificity 91%, LR = 4.4).

IgG autoantibodies against A1-23 were not correlated to any clinical parameters obtained from the patients.

IgG antibodies against A120-165 were associated with DAS28 (two-tailed *p* = 0.04, *r* = 0.2598, 95% confidence interval 0.0053–0.4827) and ESR (two-tailed *p* = 0.04, *r* = 0.3288, 95% confidence interval 0.083–0.537) in patients with RA using spearman correlation.

Using multivariate analysis, the number of tender joints (*p* < 0.001) and swollen joints (*p* < 0.001) were significantly higher in patients with RA and IgG anti-p29ING4 A120-165 than in patients with RA and without anti-p29ING4 A120-165.

IgA autoantibodies against A120-165 were associated with DAS28 (*p* = 0.002, *r* = 0.3880, 95% confidence interval 0.148–0.585), tender joint count (*p* = 0.003, *r* = 0.3780, 95% confidence interval 0.134–0.579), CRP (*p* = 0.004), and ESR (*p* = 0.004, *r* = 0.355, 95% confidence interval 0.110–0.559) in patients with RA using Spearman’s correlation. Using multivariate analysis, tender joints were only significant different between IgA anti-p29ING4 negative and positive patients with RA (*p* = 0.042). In other groups including FID, we could not observe any correlation considering acute phase reactants or disease activity.

### ELISA detecting immune complexes of IgG or IgG1 and p29ING4

IgG p29ING4 ICs were found in 31% (11/36) of CRPS patients but only in 5% (1/22) of the trauma controls (*p* = 0.02, likelihood ratio 6.72) (see Table 1). The IgG1 p29ING4 ICs were present in 39% (14/36) of CRPS patients, and only in 9% (2/22) of the trauma controls (*p* = 0.02). Combined testing of IgG and IgG1 IC increases the sensitivity to 44% (16/36) in CRPS and to 14% (3/22) of trauma controls (*p* = 0.02, likelihood ratio 3.14).

The frequency of p29ING4 IC did not significantly differentiate between CRPS and inflammatory rheumatic diseases (Table 1).

**Fig. 1:**
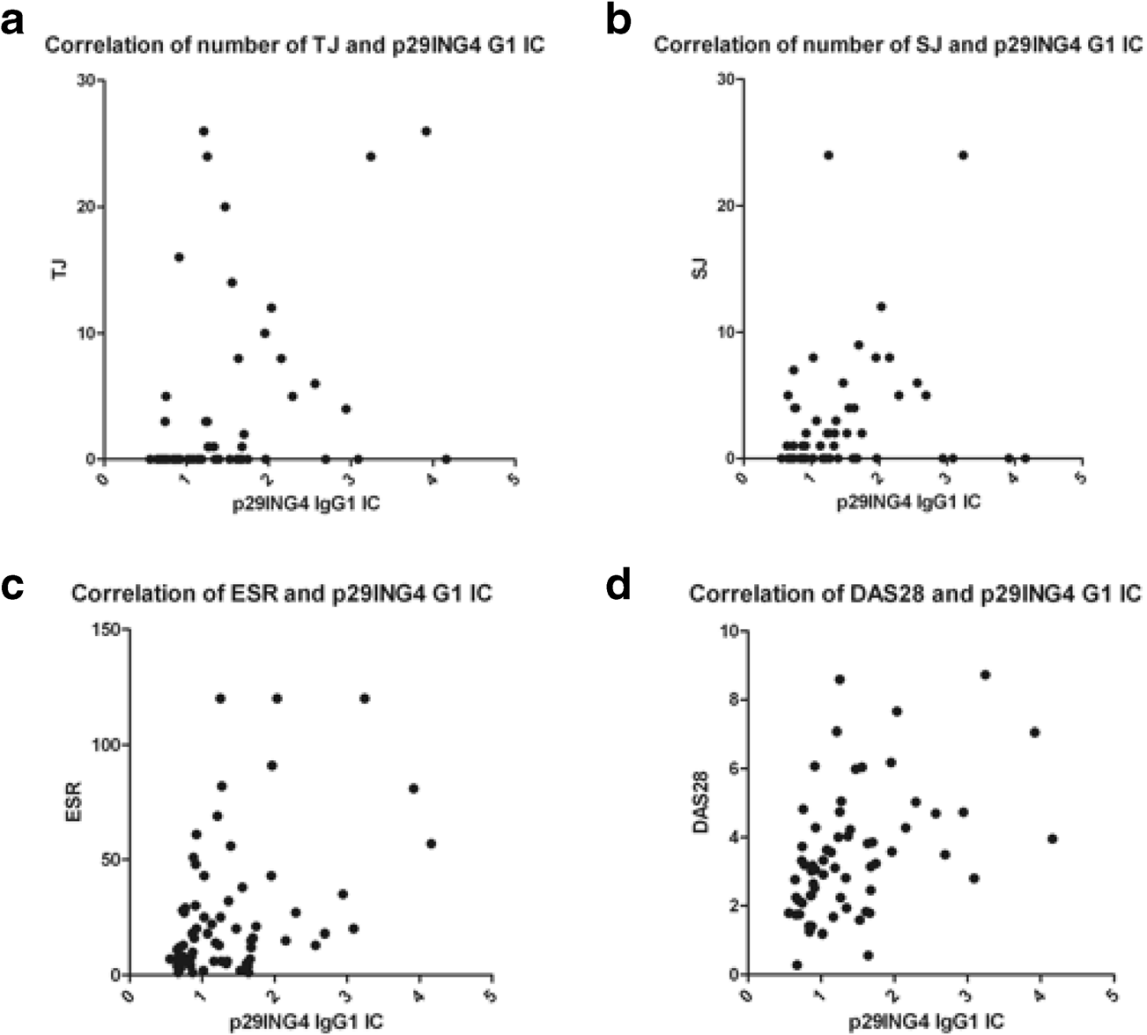
**a** Spearman’s correlation between the number of tender joints (TJ) and p29ING4 IgG1 IC. **b** Spearman’s correlation between the number of swollen joints (SJ) and p29ING4 IgG1 IC. **c** Spearman’s correlation between the erythrocyte sedimentation rate (ESR) and p29ING4 IgG1 IC. **d** Spearman’s correlation between the disease activity score 28 (DAS28) and p29ING4 IgG1 IC

RA patients with p29ING4 IgG1 IC have a significantly higher DAS28 (*p* = 0.025). Spearman’s correlation showed a significant correlation between DAS28, number of swollen joints (*p* = 0.001, *r* = 0.488, 0.2698–0.6580), number of tender joints (*p* = 0.04), VAS (*p* < 0.0001, *r* = 0, 0–0), CRP (*p* = 0.003), and ESR (*p* = 0.008) (see Fig. 1). However, there was no correlation between CRP or ESR and p29ING4 G1 IC in the other groups. Especially, all patients with CRPS had no elevated CRP values.

No significant associations were observed in consideration of p29ING4 IgG IC in patients with axSpA.

In PsA, none of the patients with p29ING4 IgG ICs received TNFi treatment (0/7 versus 14/22, *p* = 0.0063 using Fisher’s exact test).

FID with chronic infection had a higher frequency of IgG1 p29ING4 ICs than FID without chronic infection (IgG1 p29ING4 *p* = 0.029).

## Discussion

No diagnostic marker for type 1 CRPS has been established yet; therefore, it remains a clinical diagnosis based on case history and physical examination and is supported by X-ray and three-phase-bone-scintigraphy [4]. This implicates the need for diagnostic procedures to exclude other diseases. Furthermore, the diagnosis may be delayed, which may impair the prognosis of CRPS [25]. Therefore, we tried to identify biomarkers for the diagnosis of early-stage type 1 CRPS. We could identify p29ING4 as an antigen in CRPS.

In the past, several autoantibodies have been described in CRPS patients, suggesting that there may be an autoimmune pathogenesis. According to Korr et al., 30–40% of CRPS patients had autoantibodies against inducible autonomic nervous system autoantigens [26]. It is unclear whether the autoantibodies are already present before the clinical onset of CRPS. These autoantibodies were absent in controls (healthy controls, non-inflammatory neuropathy, peripheral nerve lesions without CRPS) indicating that a trauma or nerve lesion alone does not induce specific autoimmunity. Furthermore, Goebel et al. proposed that CRPS constitutes a prototype of a new kind of autoimmunity, which was termed “IRAM” (injury-triggered, regionally restricted autoantibody-mediated autoimmune disorder with minimally destructive course) [6].

Interestingly, we found p29ING4 IC in all the inflammatory joint disorders. P29ING4 IC was significantly increased in CRPS, RA, PsA, and axSpA in comparison with FID. Antibodies against p29ING4 could efficiently differentiate between CRPS and trauma control, but they are of no use considering FID.

Especially in RA, different test methods correlated with disease activity, which is quite noteworthy, because the study was not designed for evaluation of p29ING4 antibodies as disease activity markers in RA.

p29ING4 can be citrullinated by peptidyl arginine deaminase 4 (PAD4), which leads to dislinkage between p53 and p29ING4 [27]. It has been well described that PAD4 is induced by smoking in RA and could be a key factor in the etiology of RA [28, 29]. However, citrullinated p29ING4 has not been characterized as a target of autoantibodies yet. Besides, citrullination and/or PAD4 have not been described in context with CRPS.

Despite the occurrence of antibodies against p29ING4 in other inflammatory diseases, p29ING4 autoantibodies might advance the early diagnosis of p29ING4. PsA, SpA, and RA can be easily discriminated by patient’s history, biomarkers, and imaging.

## Conclusion

P29ING4 antibody is a marker of joint inflammation and early-stage type 1 CRPS. The p29ING4 autoantibodies showed 39% frequency ranging between 17 and 42% in CRPS and 0–9% in patients with distal radial fractures without CRPS. Due to the current lack of other biomarkers of CRPS, it could be helpful in its diagnostic work-up.
